# Screening Sleep Disordered Breathing in Stroke Unit

**DOI:** 10.1155/2014/317615

**Published:** 2014-05-27

**Authors:** Kirsi Väyrynen, Kati Kortelainen, Heikki Numminen, Katja Miettinen, Anna Keso, Mirja Tenhunen, Heini Huhtala, Sari-Leena Himanen

**Affiliations:** ^1^Department of Clinical Neurophysiology, Medical Imaging Centre and Hospital Pharmacy, Tampere University Hospital, 33521 Tampere, Finland; ^2^Department of Neuroscience and Rehabilitation, Tampere University Hospital, 33521 Tampere, Finland; ^3^School of Medicine, University of Tampere, 33520 Tampere, Finland; ^4^School of Health Sciences, University of Tampere, 33520 Tampere, Finland

## Abstract

In acute stroke, OSA has been found to impair rehabilitation and increase mortality but the effect of central apnea is more unclear. The aim of the present study was to evaluate the feasibility of using limited ambulatory recording system (sleep mattress to evaluate nocturnal breathing and EOG-electrodes for sleep staging) in sleep disordered breathing (SDB) diagnostics in mild acute cerebral ischemia patients and to discover the prevalence of various SDB-patterns among these patients. 42 patients with mild ischemic stroke or transient ischemic attack were studied. OSA was found in 22 patients (52.4%). Central apnea was found in two patients (4.8%) and sustained partial obstruction in only one patient (2.4%). Sleep staging with EOG-electrodes only yielded a similar outcome as scoring with standard rules. OSA was found to be common even after mild stroke. Its early diagnosis and treatment would be favourable in order to improve recovery and reduce mortality. Our results suggest that OSA can be assessed by a limited recording setting with EOG-electrodes, sleep mattress, and pulse oximetry.

## 1. Introduction

Sleep disordered breathing (SDB) is common in cerebrovascular disease [1]. In particular, obstructive sleep apnea (OSA) constitutes a risk factor for cerebrovascular events, and it is frequently seen in patients with both stroke and transient ischemic attack (TIA) [1, 2]. OSA is known to increase stroke mortality and it worsens the outcomes of rehabilitation [3–5]. Continuous positive airway pressure (CPAP) treatment for OSA in stroke patients has been found to decrease mortality, improve functional recovery, increase subjective well-being and mood, and inhibit recurrent strokes [6–10]. The effect of central apneas on mortality and functional recovery in stroke patients is less studied, but in one study central apnea did not increase mortality after stroke as OSA did [11].

Even though routine screening of OSA in the stroke units is becoming more common, it is not usually performed in the stroke units in Finland. One reason for this might be that patients with both stroke and OSA do not present the typical clinical picture of sleep apnea; they are often neither obese nor sleepy [4, 12, 13]. In addition, it might be that sleep studies are considered cumbersome in acute hospital wards. Patients in stroke units may have many different monitoring devices, as well as nasal supplementary oxygen therapy, and performing nocturnal polygraphy with additional sensors is perhaps not favored.

Sleep mattresses provide a noninvasive means to diagnose SDB. Both the SCSB (static charge sensitive bed) and the Emfit movement sensor have been proven to be suitable in detecting sleep apnea [14, 15]. SDB diagnostics with the movement sensor is patient-friendly, and the manual scoring procedure, performed in 3 min epochs, is easy and rapid. However, in limited polygraphy settings without EEG, the apnea-hypopnea index (AHI) tends to be lower than in full polysomnography (PSG) recordings [16]. In acute wards patients often sleep poorly due to the nursing and treatment time schedules. Therefore some estimation of total sleep time would be beneficial.

With this study we aim to discover the prevalence of obstructive and central sleep apnea as well as prolonged partial obstruction, among mild stroke and TIA patients in Finland. Prolonged partial obstruction is associated with continuous increase in respiratory resistance without apneas or hypopneas [17–19]. It is easily detected by the SCSB and the Emfit sensors, and its prevalence in stroke patients has not been previously studied.

In addition we aim to determinate whether limited polygraphy consisting of movement sensor and pulse oximetry in order to evaluate nocturnal breathing combined with electrooculography-signal (EOG) in order to evaluate sleep time will provide a diagnostic means of adequate quality in detecting SDB.

## 2. Materials and Methods

Patients were recruited from the Stroke Unit of Tampere University Hospital between May and October, 2010. All patients were hospitalized due to ischemic cerebral stroke or transient ischemic attack (TIA). The initial eligibility criteria for the study were as follows: (a) TIA or mild ischemic stroke, the severity defined as National Institutes of Health Stroke Scale (NIHSS) score < 12, and (b) no previous CPAP treatment for OSA. The researcher evaluated the NIHSS score at the ward and the following demographic data were gathered: age, sex, BMI, neck circumference, previous diseases, smoking, use of alcohol, subjective sleep problems (snoring and apnea) in the weeks before the stroke, and increased daytime sleepiness. For sleepiness the following question was presented: “were you sleepier than usual a couple of days before the stroke?”

The study was approved by the Ethical Committee of the Pirkanmaa Hospital District and all the subjects gave their written informed consent. If OSA was found, the subject was referred to the Sleep Center to evaluate the need for CPAP treatment. The pulmonologist then began CPAP treatment according to the standards of our hospital.

### 2.1. Recording Montage

The polygraphic recordings were performed during the first night in the hospital, whenever possible. The recordings were made in the stroke unit or at the neurologic ward. The limited polygraphy setting consisted of two EOG channels (EOG P8-A2, EOG P18-A2), electrocardiography, pulse oximetry, and the Emfit sensor (32 cm × 62 × 0.4 cm, placed under the thoracic area of the patient). In 14 randomly selected patients nocturnal sleep quality and breathing were assessed with full polysomnography, which consisted of the same sensors as the limited polygraphy and additionally six EEG channels (F3-A2, C3-A2, O1-A2, F4-A1, C4-A1, and O2-A1), submental and anterior tibialis muscle electromyography, thoracic and abdominal respiratory movements by inductive belts, and position. Airflow was measured with a thermistor and also with a nasal pressure transducer if the patient did not have nasal supplementary oxygen therapy. A sampling rate of 2 Hz was used for pulse oximetry, 10 Hz for respiratory movements, 500 Hz for ECG, and 200 Hz for the Emfit sensor and the other signals.

### 2.2. Visual Analysis

The full polysomnographies (PSG) were classified into the sleep stages according to the standard criteria. The apnea-hypopnea index (AHI) was calculated as the number of obstructive apneas and hypopneas (hypopnea rule 4b in [20]) per hour of sleep. Arousals were scored according to the criteria of the ASDA [21]. To evaluate the feasibility of the intended EOG sleep staging we classified the full polysomnographies into the sleep stages by means of the EOG-signals, without EEG or EMG, too [22]. The amounts of sleep and sleep stages comprised by the two different methods were then compared.

The limited polygraphs (POL) were classified into the sleep stages utilizing only the EOG signals. The respiratory analysis of the limited polygraphs was performed by the Emfit signal. Emfit signal was scored into one of ten categories in 3 min epochs as in our previous work [15, 19]. The mattress signal categories used were as follows: normal breathing (NB), periodic breathing type 1 (P1), obstructive periodic breathing types 1–3 (OP1–3), central periodic breathing (CP), prolonged partial obstruction (increased respiratory resistance, IRR), large movement artifacts lasting >40 s (M), epochs with at least four short periodic movements in all channels without respiratory variation (periodic movements, PM), and wake epochs (W) with EEG-defined wakefulness for more than 50% of time. The wakefulness epochs were not included in the further analyses. The respiratory results of the mattress breathing analyses are presented in percentages of total sleep time derived from the EOG scoring.

The EEG scorings were performed by two clinical neurophysiologists. The median scoring agreement was 84.1% (76.4–86.7%) with the median Kappa value being 0.71 (range 0.67–0.81). The consensus scorings were used in the final analyses. The further analyses of the recordings were performed from the first sleep onset after 8 PM to the final awakening in the morning. Obstructive AHI ≥ 15/h was selected to designate marked obstructive sleep apnea. Based on our previous study an obstructive AHI of 15/h corresponds well with obstructive periodic breathing time (%OP1 + %OP2 + %OP3) of 21% in Emfit scoring [15]. Thus the cut-off values of obstructive AHI ≥ 15/h and obstructive periodic time percentage of ≥21 were selected to define marked OSA and such patients were classified into the OSA group. The same cut-off values were used to define the central apnea group (central AI ≥ 15/h or central periodic breathing of ≥21%). The third group was comprised of the patients having a prolonged partial obstruction (IRR) percentage ≥21%, with both obstructive AHI and central AI less than 15/h (IRR group). The remaining patients composed the non-SDB group.

### 2.3. Statistics

In the statistical comparisons, nonparametric tests were used, since all the variables were not normally distributed. The comparison between the two sleep staging procedures (standard scoring versus EOG scoring) was performed by Spearman's correlation coefficient and Cohen's Kappa-analysis [23]. Pair-wised comparisons of related parameters were conducted by using the Wilcoxon test. The patient groups were compared with the Mann-Whitney test. The demographic data (sex, age, BMI, previous depression, previous stroke, previous heart failure, hypertension, hypercholesterolemia, diabetes mellitus, hypothyreosis, asthma, COPD, previous other lung diseases, use of tobacco, use of alcohol, NIHSS-score, snoring, witnessed apneas, and increased sleepiness) were evaluated with the binary logistic regression analysis. In statistical analyses *P* values < 0.05 were considered statistically significant.

## 3. Results

Altogether 46 patients volunteered for the study. Fourteen patients were recruited for full polysomnography but only twelve recordings were completed (86%). One patient wanted the sensors to be detached soon after starting the recording, and one recording terminated unexpectedly at 11 p.m. Thirty-two patients were examined by using the limited polygraphy. Two of these recordings (6%) were excluded from the analyses; one patient wanted the recording to be stopped just before falling asleep and in one recording both the Emfit and pulse oximetry signals were missed. In total, 42 patients were accepted into the analyses. The polysomnography group (PSG) consisted of one female patient and eleven male patients, whereas in the limited polygraphy (POL) group, 17 patients were female and 13 were male. The analysis start times varied between 20 : 06 and 02 : 45, with median of 21 : 19.

**Figure 1:**
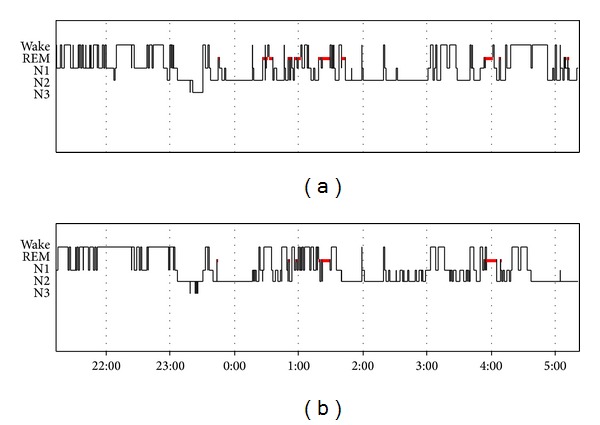
Example of the hypnograms of the same patient derived by the two different sleep staging procedures. The upper hypnogram represents standard sleep staging and the lower hypnogram represents EOG scoring. The hypnograms resemble each other but there are differences in both sleep efficiency (69% by standard sleep staging, 59% by EOG-scoring) and the arrangement of the sleep stages. The patient had moderate OSA (AHI 18.8/h).

In order to ensure the feasibility of the sleep stage scoring based on the EOG electrodes, we compared the sleep stage results derived by using the two scoring methods in the PSG group. The sleep staging agreement between the standard scoring method and the EOG scoring was 82.2% (median, range 56.3–88.2%). The median Kappa value was 0.72 (0.41–0.80), indicating substantial agreement [23]. The total sleep time (TST) estimated by the EOG scoring correlated well with the TST measured by standard scoring (Spearman's correlation coefficient 0.964). The sleep stage parameters are presented in Table 1. The total amount of sleep was higher by the EOG scoring than by the standard scoring (353.5 min versus 321 min, resp., Table 1), but the difference remained at the edge of statistical significance. The percentages in the amounts of various sleep stages between the two scoring procedures did not yield to statistically significant differences.  Figure 1 depictures the hypnograms with the lowest sleep staging agreement between the standard scoring and the EOG scoring (56.3%, Kappa 0.41 = moderate concordance).

The sleep stage parameters of all 42 patients are presented in Table 2. The median sleep efficiency, as well as the amounts of deep sleep and REM-sleep, was in general quite low even if most of the subjects slept subjectively well or fairly well during the study night (45% and 36%, resp.). Seventeen percent of the patients slept poorly and one patient (2%) felt that he had not slept at all (his total sleep time was 3 h).

Eight out of the twelve subjects in the PSG group had AHI ≥ 15/h (one female, 7 men). In the POL group 14 patients had obstructive periodic breathing ≥21% of TST (8 female, 6 male). The respiratory parameters of the PSG group and the POL group are presented in Table 3. We also calculated the AHI for sleep period time in the PSG group (sleep period time, time from sleep onset to the final awakening with wakefulness time inside the sleep period included). The AHI for the sleep period time was significantly lower than the AHI derived from TST (13.3/h, range 0.5–48.5, versus 18.1/h range 1.2–67.9, *P* value 0.002). While eight patients out of twelve had AHI > 15/h when TST was applied, only three patients had AHI > 15/h if sleep period time was used.

Altogether 17 subjects (40.5%, 9 males, 8 females) did not have sleep disordered breathing (non-SDB group, Table 4) when the PSG and the POL groups were pooled. The remaining patients had either obstructive or central breathing disorder; no one had both abnormal obstructive and abnormal central indices. OSA was found in 22 patients (52.4%, OSA group). Nine of these patients were female (41%). Central apnea was found in two male patients (4.8%, central group). IRR was found only in one female patient (2.4%, IRR group). In general the study subjects had very mild stroke as assessed by the NIHSS and they were not very overweight. Statistical comparisons were made between the non-SDB and the OSA groups only as the central group and the IRR group remained so small. There were only few statistically significant differences between the non-SDB and OSA groups; the subjects in the non-SDB group had more slow wave sleep (N3), and their oxygen desaturation indices (ODI4) were lower.

More demographic parameters are presented in Table 5. Hypertension, snoring, and increased sleepiness were frequent in all subject groups. The demographic variables were evaluated with the binary logistic regression analysis. Only increased sleepiness was identified as an independent predictor of the stroke in the non-SDB group with OR = 5.33 (CI 1.32–21.53).

## 4. Discussion

In the present study the prevalence of SDB among mild stroke and TIA patients was 59.5%. Our result for the prevalence of OSA (52.3%) in patients with TIA or mild stroke is in concordance with those of a previous study [24]. Only two subjects were found to have marked central apnea (4.8%), but this corresponds with the findings in a previous meta-analysis [25]. The prevalence of IRR in the present study was lower (2.4%) than the prevalence among our sleep laboratory patients (10.2%) [15]. The cut-off limit we previously used was lower (≥15%) but even if the value had been used, the number of IRR-patients in the present study would not have changed.

OSA increases sympathetic tone [26], inducing hypertension which may be a predisposing factor for stroke. However, there are many physiologic mechanisms behind the increase in blood pressure in OSA. For example, the hypoxia associated with periodic apnea augments sympathetic activity and blood pressure with an additional increase caused by repetitive arousals, restoring the upper airway patency [27–29]. IRR represents a different SDB entity since the periods with partial obstruction may last several minutes without arousals. In that way the increase in sympathetic activity induced by arousals does not emerge. In addition the prevailing airflow against resistance during IRR is capable of activating the pulmonary stretch receptors, which leads to the inhibition of sympathetic activation [30]. Thus, it might be that IRR does not constitute a marked risk factor for hypertension. Indeed, it has been presented that women with IRR have less hypertension than women in the general population [31]. It is possible that the risk of cardiovascular events of IRR patients might be reduced as compared to OSA patients. Thus the present study increases our knowledge of IRR, but its profound significance still remains somewhat obscure and requires further evaluation.

In the present study, the neck circumference measurement did not differ between the OSA group and the non-SDB group. This might be due to the fact that the BMI of our patients was not high in general. Similar results have been found earlier [13, 24]. In addition, our OSA patients did not present increased daytime sleepiness before stroke, unlike those who did not have OSA. The absence of excessive daytime sleepiness has been noted before [13]. It may be that patients with OSA do not necessarily recognize increased sleepiness due to the chronic sleepiness induced by OSA. The problem with our study is that sleepiness was not evaluated structurally, so it is not possible to draw strong conclusions.

In a previous meta-analysis the prevalence of female patients with OSA and stroke was found to be 35.4% [25]. In our study, the percentage of females with OSA was 41%. In that way, the portion of female patients is clearly larger than the prevalence of OSA in females in general [32]. It has been shown that females may present atypical SDB-related symptoms, for example, insomnia and depression [33], and the remarkable portion of female patients among the OSA + stroke patients may, in part, account for the atypical subjective complaints among these patients. It is possible that routine anamnesis as it conventionally relates to OSA does not necessarily reveal SDB in stroke patients, which stresses the need for OSA screening in stroke units.

Patients tend to sleep poorly in the hospital, and also the objective sleep quality of our patients was poor, as assessed by sleep efficiency. In most of the sleep recordings we utilized only the two EOG channels in the sleep stage scoring (EOG scoring) to achieve an estimate of TST. As a result, the total sleep time obtained by the EOG scoring correlated well with the standard scoring results. According to previous studies sleep staging by EOG signal seems relatively reliable and promising [22, 34] and because the EOG signals can be recorded by disposable electrodes, the attachment of the electrodes is easy. Our results suggest that sleep staging with EOG results in more valid diagnostic SDB outcome than conventional respiratory polygraphy without sleep staging even if the EOG scoring procedure may overestimate the amount of sleep.

Our other aim was to evaluate the feasibility of limited polygraphy with a sleep mattress in screening SDB. The clinical evaluation of mattress signal is easy and fast and, depending on the recording device used, adding different additional signals is often simple. In the present study, the percentage of successful recordings was higher in the POL group than in the PSG group, which supports the use of the limited recording montage in acute wards. If the number of recorded parameters is kept low, the system does not disturb the patient, enabling recordings for several nights when needed.

## 5. Conclusion

In the present study we concentrated on mild stroke and TIA patients only, and the recordings were made soon after the stroke. In general, patients with TIA and mild stroke recover well, but, according to the current knowledge, they have an increased risk of stroke recurrence [35]. As recurrent stroke might be inhibited by the proper treatment of SDB [6], screening for SDB should be favoured in the stroke units. Our results suggest that limited and less disturbing recording setting is sufficient to reveal OSA in stroke units.

## Figures and Tables

**Table 1:** Sleep stage parameters of the PSG group with the two different scoring systems.

	Standard scoring	EOG scoring	*P* value
	Median (min–max)	Median (min–max)	
TST (min)	321 (48.5–506.5)	353.5 (49.5–498.5)	0.051
SEI (%)	72.2 (42–90.9)	69.1 (42.9–89.4)	0.48
% N1	12.8 (6.5–41.2)	15.4 (6.8–48.5)	0.068
% N2	69.5 (36.1–83.2)	66.2 (37.4–82.4)	0.33
% N3	7.0 (0–23.6)	5.9 (0–17.7)	0.17
% REM	11.2 (0–22.3)	12.9 (0–25.4)	0.61

Standard scoring: sleep stage scoring according to the standard rules; EOG scoring: sleep stage scoring based on the two EOG (electrooculography) channels only; TST: total sleep time; SEI: sleep efficiency index referred to analysis time; % N1–N3: percentage of N1–N3 sleep referred to TST; % REM: percentage of REM sleep referred to TST. Statistical comparisons with the Wilcoxon test.

**Table 2:** The sleep quality parameters of the 42 patients.

	Median	Min	Max
TST (min)	368.5	48.5	510.5
SEI (%)	70.8	27.5	97.6
% N1	8.8	1.8	41.2
% N2	46.9	3.3	83.2
% N3	6.5	0	26.2
% REM	7.1	0	31.2

TST: total sleep time; SEI: Sleep Efficiency Index referred to analysis time; % N1–N3: percentage of N1–N3 sleep referred to TST; % REM: percentage of REM sleep referred to TST.

**Table 3:** The respiratory parameters of the PSG group and the POL group.

	PSG group			POL group					
	Median	Min	Max	Median	Min	Max
AHI obstructive (*n*/h)	18.1	1.2	67.9			
AHI central (*n*/h)	0	0	1.2			
ODI4	4.5	0	32.5	6.0	0	64.8
% OP1–OP3	27.2	2.6	69.3	14.1	0	45.8
% CP	0	0	4.7	0	0	51.6
% IRR	0.8	0	9.6	0.5	0	25.4
SaO_2_min (%)	87.5	79.0	93.0	85.0	75.0	91.0
SaO_2_mean (%)	93.7	90.7	96.5	93.2	89.5	97.1
Pulse (bpm)	55	46	73	58	46	86

AHI obstructive: number of obstructive apneas and hypopneas per hour of sleep; AHI central: number of central apneas per hour of sleep; ODI4: number of desaturations per hour of sleep; % OP1–OP3: percentage of time with obstructive periodic breathing as detected by Emfit sensor; % CP: percentage of time with central periodic breathing as detected by Emfit sensor; % IRR: percentage of time with prolonged partial obstruction as detected by Emfit sensor; SaO_2_min: minimum oxygen saturation percentage; SaO_2_mean: average oxygen saturation percentage; Pulse: mean pulse per minute.

**Table 4:** Some demographic and sleep parameters of the different subject groups.

	Non-SDB, *n* = 17	OSA, *n* = 22	Central, *n* = 2	IRR, *n* = 1	Non-SDB versus OSA
	Median (min–max)	Median (min–max)	Median (min–max)	Value	*P* value
BMI	26.0 (19.9–32.7)	26.6 (21.9–37.4)	25.3 (25.2–25.3)	27.5	0.705
AGE	62 (45–79)	69 (37–82)	77 (75–78)	68	0.131
TST	426 (49–511)	321 (105–490)	300 (282–318)	416	0.116
SEI	72.8 (42.0–97.6)	70.3 (27.5–95.7)	65.2 (61.3–69.1)	67.2	0.236
% N1	8.9 (2.6–41.2)	9.0 (1.8–23.0)	12.5 (11.9–13.1)	8.2	0.962
% N2	45.9 (33.1–73.9)	57.8 (3.3–83.2)	44.7 (42.7–46.7)	53.6	0.168
% N3	12.4 (3.3–26.2)	4.3 (0–23.6)	1.5 (1.1–1.8)	2.4	0.005
% REM	6.9 (0–22.3)	10.8 (1.0–31.2)	6.9 (5.2–8.5)	3.1	0.539
ODI4	1.8 (0–8.2)	16.4 (0–64.8)	9.7 (4.2–15.1)	0.7	0.000
SaO_2_min (%)	87 (80–91)	85 (75–93)	87 (84–89)	85	0.053
SaO_2_mean (%)	93.6 (90.7–97.1)	93.3 (89.5–96.8)	94.5 (93.3–95.7)	91.7	0.975
Pulse (bpm)	61 (50–86)	58 (46–78)	51 (50–52)	51	0.133
NIHSS	2 (0–6)	1 (0–4)	3 (2–4)	1	0.138
Neck (cm)	40 (34–44)	41 (38–53)	41 (40–42)	39	0.231

NIHSS: National Institutes of Health Stroke Scale; Neck: neck circumference in centimeters.

The rest of the abbreviations are as in Tables 1 and 3. Statistical comparisons between the Non-SDB group and the OSA group with the Mann-Whitney test.

**Table 5:** Demographic parameters of the 42 patients.

	Non-SDB	OSA	Central	IRR
Depression (%)	5.9	4.5	0	0
Previous stroke (%)	5.9	13.6	0	0
Heart failure (%)	23.5	27.3	0	0
Hypertension (%)	47.1	63.6	50	100
Hypercholesterolemia (%)	11.8	13.6	0	0
Diabetes mellitus (%)	5.9	4.5	0	0
Hypothyreosis (%)	5.9	4.5	0	100
Asthma (%)	5.9	0	0	0
COPD (%)	17.6	4.5	0	0
Tobacco use (%)	47	18.1	50	0
Alcohol use (%)	29.4	22.7	0	0
Snoring (%)	52.9	54.5	50	100
Witnessed apneas (%)	5.9	22.7	50	0
Increased sleepiness	76.5	40.9	0	100

COPD: chronic obstructive pulmonal disease.
